# Honesty requires time—a reply to Foerster et al. (2013)

**DOI:** 10.3389/fpsyg.2013.00634

**Published:** 2013-09-26

**Authors:** Shaul Shalvi, Ori Eldar, Yoella Bereby-Meyer

**Affiliations:** Department of Psychology, Ben-Gurion University of the Negev, Beer Sheva, Israel

**Keywords:** honesty, ethics, morality, decision making, judgment and decision making, justifications, cheating

*“Focusing on tempting situations in which cheating allows serving one's self-interest, we suggest that cheating is an automatic tendency and that the need for justification matters only when people have time to deliberate”* (Shalvi et al., 2012; p. 1264). Foerster et al. (2013) challenged this proposition. Here, we review their critique and propose that their findings do not contradict, but rather support our observation—that honesty requires time.

In Shalvi et al. (2012), we studied whether in tempting, private situations, time-pressure induces self-serving behaviors—specifically, lying for profit. Using a simple die-under-cup task [(Shalvi et al., 2011a, b; based on Fischbacher and Föllmi-Heusi (2013)], we asked participants to roll a die under a paper cup (ensuring privacy), report the observed number, and earn money with higher numbers leading to higher pay. Since only participants observed the die roll, they could lie to boost profit. We manipulated the time participants had to report their outcome. In two experiments, time-pressure led to higher aggregated reports than reporting without time-pressure. We suggested that time-pressure leads people to act upon their self-serving desires, and only with time they calibrate to what is considered normative (honest) behavior. Recent independent work using the deception game (Gneezy, 2005) revealed a similar pattern: contemplating led to less deception (Gunia et al., 2012).

Foerster et al. (2013) challenged our work both theoretically and methodologically. Challenging our theoretical proposition, they listed research suggesting lying is more cognitively demanding than honesty (e.g., Spence et al., 2001; Walczyk et al., 2009; Debey et al., 2012). This important line of work is interesting and makes sense. When participants are asked to lie or tell the truth regarding information they read, it is reasonable that lying requires more cognitive effort. Our work does not challenge this line of work, rather, it complements it. Unlike the cited papers, we focused on situations in which lying is *self-serving*. This is fundamentally different from lying without a motivation to do so. Motivation is a strong predictor of human behavior. We suggest that in *tempting*, private settings, and when the lie is simple to craft, time pressure induces self-serving behaviors—even lies.

Foerster et al. further challenged certain aspects of our die-under-cup task suggesting: “As a central feature of the die under cup paradigm, participants can—in principle—generate their response before actually rolling the die” (p. 473). However, if our participants indeed generated their responses before rolling, one should not expect any differences in reported outcomes between conditions. If participants decided what to report before rolling, they should have lied to the same extent regardless of being instructed to report quickly or not. This was not what we found, rendering this possibility unlikely.

To overcome the possibility that participants were generating responses before rolling Foester et al. modified our task by varying *“the time available for reflection about the (dis)honesty of the reports on two levels: individual die rolls and blocks of rolls”* (p. 473). Participants were instructed to report the outcome of an 8-sided die in 12 different rolls determining their pay. The block level time manipulation included six rolls before and six rolls after a break. This time manipulation, rolling before and after the break, replicated our observation that honesty requires time (Gunia et al., 2012; Shalvi et al., 2012). Foerster et al. only found evidence for lying in the first block of die rolls, not in the second block.

Foerster et al. using an individual die roll manipulation, obtained results that seem contradictory to ours. In the *immediate* trials, participants reported the outcomes of three die rolls immediately after rolling each of them. In the *delayed* trials, participants observed the outcome of the roll which they should report, but reported this outcome after shaking the die-under-cup another time. This manipulation was employed both before and after the break. Before the break, participants lied more in the *delayed* compared to the *immediate* trials. While interesting, these conditions do not differ from each other on the key parameter namely, time pressure. Specifically, the time provided to report was not manipulated nor measured. It is unclear if participants took more time to report their outcomes in the delayed condition. Moreover, the delayed trials required participants to report the die roll outcome after engaging in another die roll, while the immediate trials did not. Engaging in the extra roll may increase lying for two reasons: (1) it may disrupt deliberation, and/or (2) attract attention to the potential desired outcomes (i.e., high numbers) participants were generating by rolling again. It is difficult to interpret what was driving participants' behavior. We believe that this manipulation does not provide direct evidence to the role of time in shaping dishonesty. Furthermore, it is unclear how it addresses the possibility of generating responses before rolling.

## Conclusion

In Shalvi et al. (2012) we employed a single-shot, die-under-cup task to study people's dishonesty under time-presure. Participants reported the outcome of a 6-sided die, rolled under a paper-cup, and were paid according to a simple payoff rule. We induced time pressure by asking participants to report within a short timeframe, or without such timeframe. Inducing time pressure increased aggregate dishonesty (see also Gunia et al., 2012). Foerster et al.'s (2013) multiple modifications to our original design (e.g., 8-sided vs. 6-sided die; multiple vs. one-shot decision; within vs. between-subject design) make comparing the two studies difficult. Nevertheless, the observation that in some settings the immediate decision is to be honest, seems interesting and deserves further research. In sum, the time manipulation employed by Foerster et al., reporting before vs. after a break, led to another replication of our observation. Lying was observed only at the beginning of the experiment, not at the end. Together, findings from three labs, employing different methods (Gunia et al., 2012; Shalvi et al., 2012; Foerster et al., 2013) suggest that in tempting situations, honesty requires time.
